# A Novel Injectable Borate Bioactive Glass Cement as an Antibiotic Delivery Vehicle for Treating Osteomyelitis

**DOI:** 10.1371/journal.pone.0085472

**Published:** 2014-01-10

**Authors:** Hao Ding, Cun-Ju Zhao, Xu Cui, Yi-Fei Gu, Wei-Tao Jia, Mohamed N. Rahaman, Yang Wang, Wen-Hai Huang, Chang-Qing Zhang

**Affiliations:** 1 Department of Orthopedic Surgery, Shanghai Sixth People's Hospital, Shanghai Jiao Tong University, Shanghai, People's Republic of China; 2 Department of Orthopedic Surgery, Liaocheng People's Hospital, Liaocheng, Shandong, People's Republic of China; 3 Institute of Bioengineering and Information Technology Materials, Tongji University, Shanghai, People's Republic of China; 4 Department of Materials Science and Engineering, Missouri University of Science and Technology, Rolla, Missouri, United States of America; Institute for Frontier Medical Sciences, Kyoto University, Japan

## Abstract

**Background:**

A novel injectable cement composed of chitosan-bonded borate bioactive glass (BG) particles was evaluated as a carrier for local delivery of vancomycin in the treatment of osteomyelitis in a rabbit tibial model.

**Materials and Methods:**

The setting time, injectability, and compressive strength of the borate BG cement, and the release profile of vancomycin from the cement were measured in vitro. The capacity of the vancomycin-loaded BG cement to eradicate methicillin-resistant *Staphylococcus aureus* (MRSA)-induced osteomyelitis in rabbit tibiae in vivo was evaluated and compared with that for a vancomycin-loaded calcium sulfate (CS) cement and for intravenous injection of vancomycin.

**Results:**

The BG cement had an injectability of >90% during the first 3 minutes after mixing, hardened within 30 minutes and, after hardening, had a compressive strength of 18±2 MPa. Vancomycin was released from the BG cement into phosphate-buffered saline for up to 36 days, and the cumulative amount of vancomycin released was 86% of the amount initially loaded into the cement. In comparison, vancomycin was released from the CS cement for up 28 days and the cumulative amount released was 89%. Two months post-surgery, radiography and microbiological tests showed that the BG and CS cements had a better ability to eradicate osteomyelitis when compared to intravenous injection of vancomycin, but there was no significant difference between the BG and CS cements in eradicating the infection. Histological examination showed that the BG cement was biocompatible and had a good capacity for regenerating bone in the tibial defects.

**Conclusions:**

These results indicate that borate BG cement is a promising material both as an injectable carrier for vancomycin in the eradication of osteomyelitis and as an osteoconductive matrix to regenerate bone after the infection is cured.

## Introduction

Curing osteomyelitis (bone infection) presents a clinical challenge because once infection has entered bone, it is difficult to treat. Furthermore, the infection has a significant relapse rate despite gradual advances in surgical techniques and the development of novel antimicrobial agents over many years [1], [2]. Osteomyelitis can occur at all ages and diabetic patients are particularly susceptible to the infection. Osteomyelitis is also becoming more common in orthopedic surgery as the number of hip and knee joint replacements increases with an aging population. The treatment for osteomyelitis includes removing the infected areas of bone followed by months of extensive intravenous antibiotic treatment.

Local delivery of high doses of antibiotics is desirable in treating osteomyelitis because it can facilitate the delivery of antibiotics by diffusion to avascular areas that are inaccessible by systemic antibiotics while minimizing potential systemic toxicity [3]. In some cases, infecting organisms that are resistant to drug concentrations achieved by systemic antibiotics are susceptible to the higher drug concentrations provided by local antibiotic delivery. Poly(methyl methacrylate) (PMMA), calcium sulfate (CaSO_4_), calcium phosphate ceramics such as hydroxyapatite (HA) and beta-tricalcium phosphate (β-TCP), and biodegradable polymers, synthetic or natural, such as poly(lactic acid) poly (glycolic acid), and their copolymers, collagen and chitosan) have been widely studied or used as carriers for local drug delivery [4], [5]. However, those carriers have limitations such as bioinertness (e.g., PMMA) or inadequate degradation rate (e.g., HA), poor osteoconductivity, rapid antibiotic elution rate, transient cytotoxicity, and low strength [6], [7].

In recent years, various compositions of borate glass have been investigated for biomedical applications because they are bioactive, biocompatible and osteoconductive. Borate-based bioactive glasses (BG) degrade and convert more rapidly and more completely to HA than the more widely studied silicate bioactive glasses such as 45S5 glass when placed in an aqueous phosphate solution such as body fluid [8]–[10]. Furthermore, the degradation rate of borate-based BG can be controlled by changing the B_2_O_3_ content of the glass to SiO_2_ to form borosilicate bioactive glasses [11]. Our previous studies have demonstrated that borate BG pellets can serve as a carrier for sustained delivery of antibiotics *in vitro* and *in vivo*, and for the treatment of osteomyelitis in a rabbit model [12]–[14].

Injectable biomaterials can be implanted using minimally invasive surgical techniques, resulting in less pain, shorter recovery time, and lower healthcare cost when compared to conventional surgery. They are also more convenient for filling irregular-shaped bone defects, leaving little dead space for bacterial survival. Injectable calcium sulfate (CS) loaded with antibiotics is one of the most widely studied materials [15]–[17], but it suffers from limitations such as rapid resorption and antibiotic release rates, low mechanical strength, rapid reduction in its mechanical strength with time *in vivo*, and transient cytotoxic effects leading to inflammatory reactions [18], [19].

In this study, we constructed an injectable local antibiotic delivery system, which was composed of both powder and liquid components. The powder component was borate glass. The liquid component was a solution of chitosan, citric acid and glucose. As described earlier, borate BG has several properties which make it attractive for biomedical applications. Chitosan, which has high viscosity in solution and excellent biocompatibility, is a useful carrier for controlled release of drugs because of its complexation property [20]; it can also be used as a binder to improve the elution characteristics of antibiotic carrier materials [21]. Citric acid has three carboxyl groups and one hydroxyl group, which can be cross-linked with chitosan to increase the viscosity of the liquid phase. Citric acid could also undergo a chelation reaction with calcium in borate BG, and thereby influence setting times, mechanical properties and biocompatibility of cements. Glucose which contains five hydroxyl groups could react with citric acid, and inhibit its effect. The optimal weight ratio of chitosan, citric acid and glucose was justified in our preliminary research, which we used to design cement with an appropriate setting time and mechanical properties.

The objective of this study was to evaluate borate BG cement as a carrier of vancomycin in the treatment of osteomyelitis in a rabbit tibial model. Vancomycin was used because it is a broad-spectrum glycopeptide antibiotic that is active against Gram-positive bacteria. It is used clinically to treat osteomyelitis in patients who are allergic to penicillin or who are infected with the bacterium methicillin-resistant *Stayphylococcus aureus* (MRSA) [22]. The key physico-chemical characteristics of the BG cement and the release profile of vancomycin from the cement were measured *in vitro*, while the capacity of the BG cement to eradicate osteomyelitis in rabbit tibiae at 2 months post-surgery was evaluated using radiography, microbiological testing, and histology. For comparison, the capacity of a vancomycin-loaded commercial CS cement to eradicate the osteomyelitis was also studied.

## Materials and Methods

### Ethics statement

The study protocol adhered to the recommendations of the US Department of Health for the care and use of laboratory animals and was approved by the Ethics Committee of Shanghai Jiao Tong University.

### Preparation of vancomycin-loaded borate BG cement

The injectable cement used in the present study was composed of borate BG particles bonded together with chitosan. BG with the composition (mol%) 6Na_2_O–8K_2_O–8MgO–22CaO–54B_2_O_3_–2P_2_O_5_ was prepared by conventional methods and ground to form particles of <10 µm in size, as described previously [11]. The bonding phase was prepared by mixing chitosan, citric acid and glucose in the ratio 1∶10∶20 (by weight) to form a solution. To prepare the BG cement, a mixture of vancomycin powder (Eli Lilly; Japan) and BG particles (80 mg of vancomycin per gram of BG) was added to the bonding solution (weight ratio of solid phase to liquid  = 2∶1), and the system was mixed for 1 minute in a mortar and pestle to form a paste. For comparison, an injectable putty was prepared by mixing vancomycin powder, a commercially available CS powder (Allomatrix®; Wright Medical) and the mixing solution provided with the CS powder, as recommended by the manufacturer. The same concentration of vancomycin was used in both the BG paste and the CS putty, equal to 8 wt% of the BG or CS. Prior to use, the solid starting materials were sterilized by ã-irradiation (25 kGy), and all materials were prepared under sterile conditions.

### Characteristics detection

The initial and final setting times of the vancomycin-loaded borate BG paste (formed as described above) were measured at room temperature using a standard test by means of Gillmore needles, as described in ASTM C266–99 [23]. The injectability of the paste was evaluated using an extrusion test, as described previously [24]. Briefly, after mixing, the paste was loaded into a commercial syringe with a capacity of 20 mL and an opening 2 mm in diameter. At selected times from the completion of the mixing step, the paste was extruded from the syringe (held rigidly in a fixture) by applying a force using a universal testing machine (BIONIX; MTS, Eden Prairie, MN) at a crosshead speed of 15 mm/min. The extrusion was stopped when the applied force reached 100 N. The injectability coefficient (I) was defined as
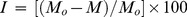
(1)where M_o_ is the initial mass of the cement loaded into the syringe, and M is the mass remaining in the syringe after extrusion. At each time point, 6 samples of the cement were tested, and the results are expressed as a mean ± standard deviation (SD).

The compressive strength of the borate BG cement was measured as a function of immersion time in phosphate buffered saline (PBS) at 37°C using a mechanical testing machine (BIONIX, MTS, MN) at a crosshead speed of 0.5 mm/min. Cylindrical samples (6 mm in diameter ×15 mm) of the as-fabricated cement (formed by allowing the BG paste to harden for 30 minutes), and the as-fabricated cement immersed in PBS for various times (12, 24, 48, 96 and 168 h) were tested. Six samples were tested at each time point and the results are expressed as mean ± SD. The surface morphology and structure of the BG cement, before and after immersion in PBS, were examined using a field-emission scanning electron microscope (SEM) with an energy dispersive X-ray spectroscopy (EDS) analysis unit attached (Quanta 200 FEG, FEI Co., The Netherlands). There is a table arrived from the EDS analysis, which showed the contents of all element presented in the sample. The Ca/P ratio was calculated from these contents of Ca and P. X-ray diffraction (XRD; Model D/max 2550 v, Rigaku International Corp., USA) (Cu Ka radiation;λ15406 nm) at a scan rate of 1.0°2θ per minute was also performed.

### Antibiotic release study

The release of vancomycin from the BG and CS cements was measured using cylindrical samples (6 mm in diameter ×15 mm) immersed in sterile PBS at 37°C. Each sample was immersed in 10 mL PBS, and the medium was collected and replaced with fresh PBS every 48 h until no vancomycin could be detected in the medium (detection limit <0.2 µg/mL). The concentration of vancomycin in the medium was measured using high-performance liquid chromatography (HPLC) as described previously [25]. The percentage of vancomycin released into the medium was determined from the measured amount of vancomycin in the medium and the amount initially loaded into the cements. In order to determine whether the antibacterial activity of vancomycin was affected by the cement preparation process, the antibacterial activity of the vancomycin eluted from the cements was compared with that for the as-received vancomycin. Testing for the minimum inhibitory concentration (MIC) of vancomycin against MRSA (ATCC43300) was performed using an antibiotic 2-fold tube dilution method described elsewhere [26], which yielded 0.125, 0.25, 0.5, 1, 2, 4, 8, 16, 32, 64 µg/mL drug concentrations in 10 tubes with 2 mL sterile cation-supplemented Mueller-Hinton broth (Oxoid). The tubes were then inoculated with MRSA at 10^7^ CFU/mL. Three no-drug tubes (not inoculated with any bacterium, inoculated with MRSA, and inoculated with standard bacterium) were also performed as control. MIC was the lowest concentration of antibiotic that prevented turbidity after 24 h of incubation at 37°C.

### Surgical procedure

Seventy adult New Zealand White rabbits (mean weight  =  2.20 kg; range  = 1.8–2.7 kg) were used in this study. Osteomyelitis was induced in the right tibia of the rabbits using the method described by Norden *et al*. [27]. Briefly, under general anesthesia with 3% sodium pentobarbital (1 mL/kg), the proximal part of the tibia was exposed and a hole was created manually through the cortex into the medullary cavity using a needle. Then 0.1 mL of 5% sodium morrhuate, 0.1 mL of MRSA suspension (1×10^8^ CFU/mL) and 0.1 mL of sterile PBS were injected sequentially into the medullary cavity. Four weeks later, the presence of osteomyelitis was evaluated from radiographs by two radiologists, independently and in a blinded manner, according to the scoring system described by Norden *et al*.

Sixty-three animals that developed osteomyelitis were chosen for subsequent studies. Five animals were sacrificed for microbiological and histological examination. The remaining animals were randomly divided into 4 groups. The animals in Group 1 (n = 10) did not receive any further treatment, while the Group 2 animals (n = 16) were treated by debridement and daily intravenous injection of vancomycin (6 mg per kg body weight) for one month; the animals in Group 3 (n = 16) were treated by debridement followed by implantation of injectable vancomycin-loaded CS cement, and the animals in Group 4 (n = 16) were treated by debridement followed by implantation of injectable vancomycin-loaded BG cement. Debridement was performed by making a cortical bone window (2.0−2.5×1.0 cm), and removing the purulent marrow by saline lavage. Samples of the debrided tissues were used to confirm MRSA infection. After debridement, the cements were injected into the medullary space of the defects to treat the infection. Then the surgical wounds were sutured and disinfected. The animals were closely monitored after surgery and normal activity was allowed.

### Blood analysis

Before surgery and at monthly intervals post-surgery, venous blood was taken from the animals and used to measure the white blood cell count (WBC), and to analyze liver and renal function, including glutamate-pyruvate transaminase (GPT), glutamic-oxal(o)acetic transaminase (GOT), urea (Ur) and creatinine (Cr). The concentration of vancomycin in the serum of animals in Groups 3 and 4 was measured as a function of implantation time using HPLC, while the boron concentration in the serum of the Group 4 animals was measured from 1 h to 7 days post-surgery using inductively coupled plasma-atomic emission spectroscopy (ICP-AES; Optima 2100DV, USA).

### Radiographic evaluation

Standard digital anterior−posterior and lateral radiographs of the right hind limbs were taken before surgery and at 2 months post-surgery. Periosteal new bone formation (PNBF), sequestral bone formation (SBF) and destruction of bone (DB) were used to evaluate the severity of osteomyelitis by two radiologists in a blinded independent manner. The radiographs were also evaluated using the scoring system described by Norden *et al*. [27]. Briefly, four radiographic parameters (presence of SBF, presence of PNBF, presence of DB and extent of involvement) were evaluated for each radiograph. A numerical score was assigned to each variable. All four scores were added together to give an overall ranking for radiographic severity. A higher score represented increased severity of osteomyelitis.

### Microbiological evaluation

The animals were euthanized by intravenous administration of pentobarbital sodium at 2 months post-surgery. Using sterile techniques, swab specimens for microbiological examination were immediately taken from the bone, the marrow and the surrounding area of soft tissues. They were inoculated on blood agar and incubated for 48 h at 37°C. Identification of *S. aureus* was performed with the coagulate tube test and the API Staph system (ATB 32Staph. Bio-Merieux, Marcy-l′Etoile, France). Resistance to methicillin was further confirmed by detection of the mecA gene using PCR.

### Histopathological evaluation and SEM analysis

After the rabbits were sacrificed, samples of the defects together with surrounding bone were taken and fixed in 10% formalin for 7 days, then decalcified in EDTA and embedded in paraffin using routine histological techniques. The embedded specimens were serially sectioned (5 µm thickness) in the original longitudinal direction and the sections were stained with hematoxylin and eosin (H&E) for histological examination. All sections were examined and evaluated for intraosseous acute inflammation (IAI), intraosseous chronic inflammation (ICI), periosteal inflammation (PI) and bone necrosis (BN) using the scoring system described by Smeltzer *et al*. [28]. Samples from the Group 4 animals (implanted with the BG cement) were also examined by SEM (Quanta 200 FEG). The tissue in the defect area was cut in the original transverse direction to give sections of 3–5 mm thickness. The specimens were fixed using 5% glutaraldehyde phosphate solution, dehydrated through a graded series of ethanol, and examined by SEM.

### Statistical analysis

All data were analyzed using the Statistical Package for the Social Sciences, Version 13.0 (SigmaStat, SPSS Science, Chicago, IL, USA), and the results were expressed as mean ± standard deviation (SD). A one-way ANOVA was performed to assess significant differences among WBC counts, liver and renal function, radiographic scores and histopathological scores of the four groups. MRSA infection rates of the four groups identified by Radiographic evaluation and Microbiological evaluation were analyzed qualitatively by chi-square test. Differences were considered to be significant at *P*<0.05.

## Results

### Characteristics of vancomycin-loaded borate BG cement *in vitro*

**Figure 1:**
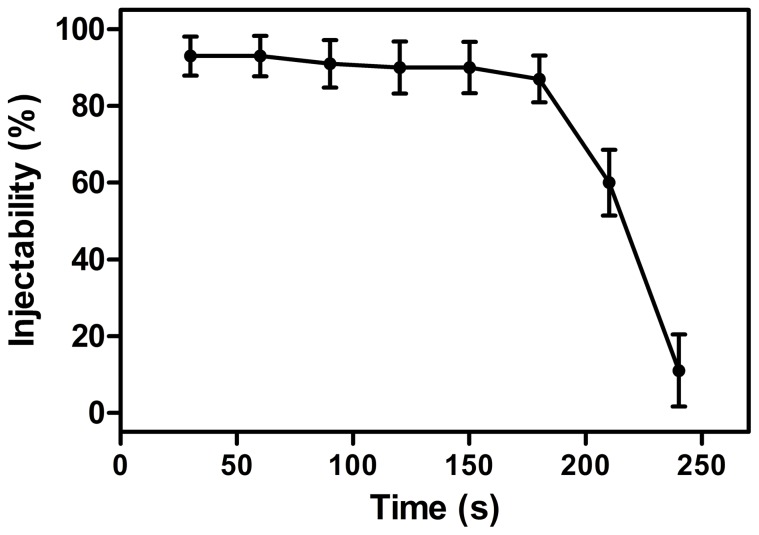
Percent injectability of vancomycin-loaded borate bioactive glass (BG) cement as a function of time after mixing.

**Figure 2:**
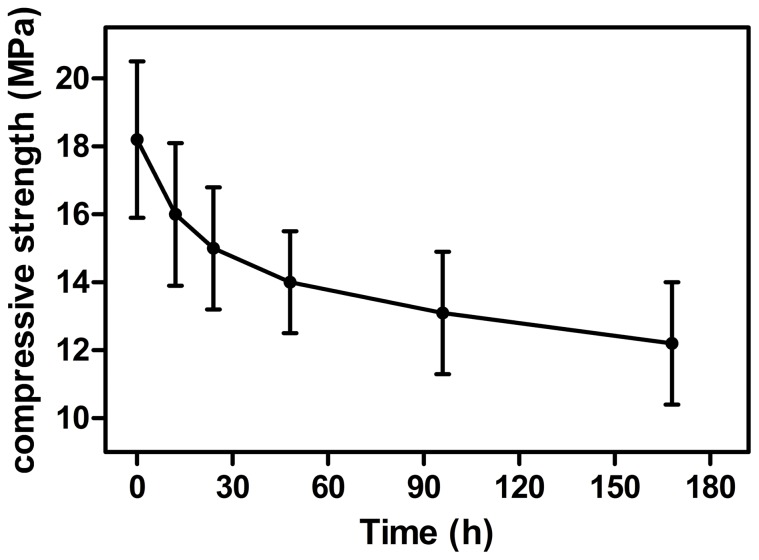
Compressive strength of vancomycin-loaded borate BG cement after hardening and as a function of immersion time in phosphate-buffered saline (PBS) *in vitro*.

**Figure 3:**
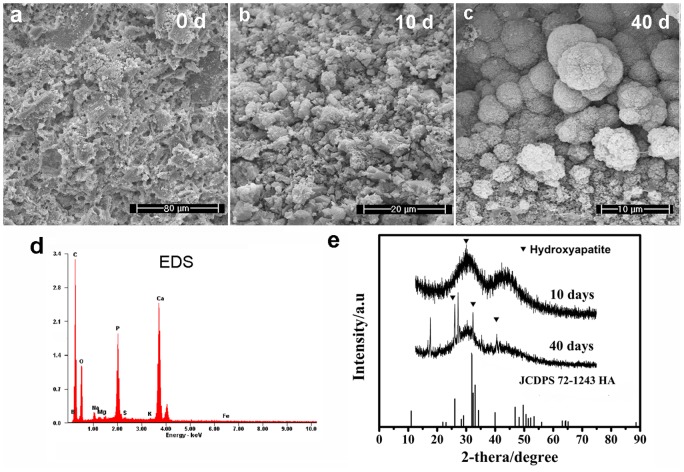
Characteristics of vancomycin-loaded borate BG cement in vitro. SEM images of the surface of vancomycin-loaded borate BG cement as fabricated (after hardening) (a) and after immersion in PBS for 10 days (b) and for 40 days (c). EDS analysis showed that particles formed on the surface of the specimens were composed of Ca and P (d). After immersion in PBS for 40 days, the XRD pattern of the samples showed similar peaks to those characteristic of reference hydroxyapatite (JCPDS 72–1243) (e).

The initial and final setting times of the vancomycin-loaded borate BG cement were 8.1±2.3 min, and 27.6±6.4 min, respectively. The percent injectability of the cement (Fig. 1) remained above 90% during the first 3 minutes after the mixing step, and decreased rapidly thereafter. As prepared, the vancomycin-loaded cement had a compressive strength of 18.2±2.3 MPa; upon immersion of the cement in PBS, the strength decreased to 15.0±1.8 MPa after 1 day and more gradually thereafter, to 12.2±1.8 MPa after 7 days (Fig. 2). SEM of the surface of the hardened vancomycin-loaded BG cement showed a heterogeneous microstructure with some pores, which might be caused by bubbles produced during the mixing process (Fig. 3a). After immersion in PBS for 10 days, a few particles, composed of Ca and P according to EDS analysis (Fig 3d), formed on the surface of the specimens (Fig. 3b). After immersion for 40 days, the precipitated particles almost covered the whole surface of the cement which appeared denser and more uniform (Fig. 3c); EDS analysis showed that the Ca/P atomic ratio of the particles was 1.60±0.22, which is close to the value for stoichiometric hydroxyapatite (1.67). The XRD pattern of the samples also showed characteristic peaks similar to reference hydroxyapatite (JCPDS 72–1243) (Fig 3e).

### Vancomycin release profile and antibacterial activity *in vitro*

**Figure 4:**
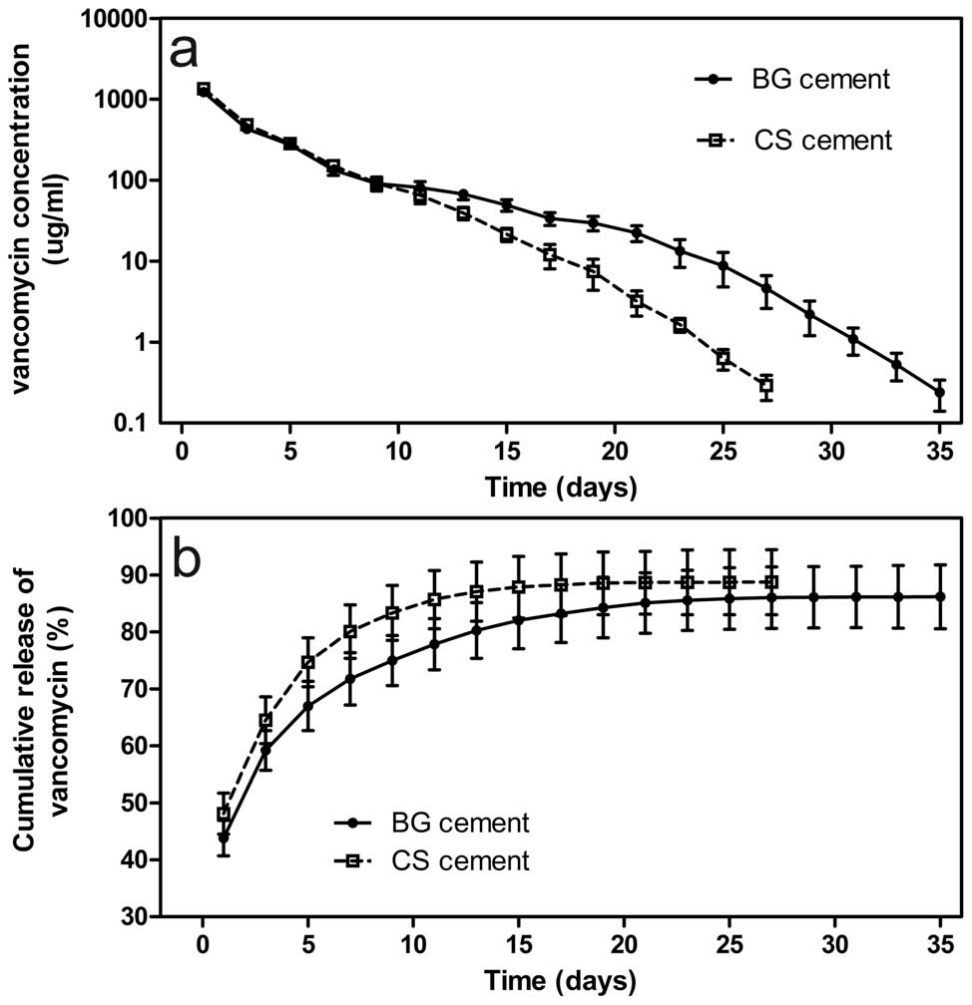
Vancomycin release profile in vitro. (a) Concentration of vancomycin released from borate BG cement and calcium sulfate (CS) cement into PBS at different time points; (b) cumulative amount of vancomycin released (as a percentage of the amount initially loaded into the cements) as a function of immersion time in PBS.

**Figure 5:**
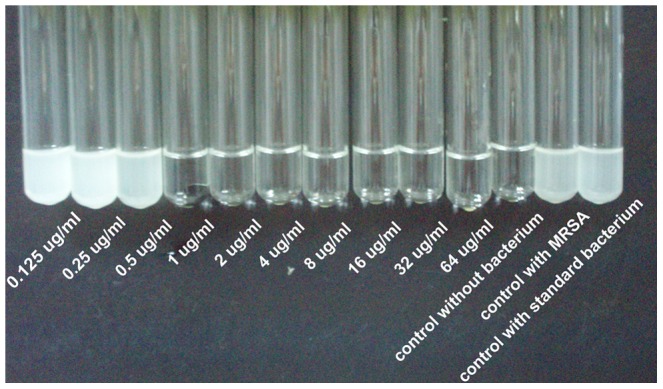
The MIC against MRSA for vancomycin was determined using the antibiotic twofold tube-dilution method. Three no-drug tubes (not inoculated with any bacterium, inoculated with MRSA, and inoculated with standard bacterium) were also performed as control. MIC was the lowest concentration of antibiotic that prevented turbidity after 24 h of incubation at 37°C. In this study, the MICs of all three groups (non-processed vancomycin, vancomycin released from borate BG cement and CS cement) were 1 µg/mL.

The release profiles of vancomycin from the BG cement and the CS cement into PBS (Fig. 4) showed a more rapid release during the first 2 days, followed by a slower, more sustained release thereafter. The cumulative amount of vancomycin released from the BG cement at 2 days was 44% of the total amount initially loaded into the as-prepared cement, compared to 48% for the CS cement. At day 36, the cumulative amount of vancomycin released from the BG cement was 86%, after which no measurable release was observed (detection limit <0.2 µg/mL). In comparison, the cumulative amount of vancomycin released from the CS cement was 89% at day 28, after which no further release was detected. The MICs of all three groups (non-processed vancomycin, vancomycin released from borate BG cement and CS cement) were 1 µg/mL, indicating that the cement preparation process did not affect the antibacterial activity of the vancomycin (Fig. 5).

### Blood analysis of animals implanted with vancomycin-loaded borate BG cement

**Figure 6:**
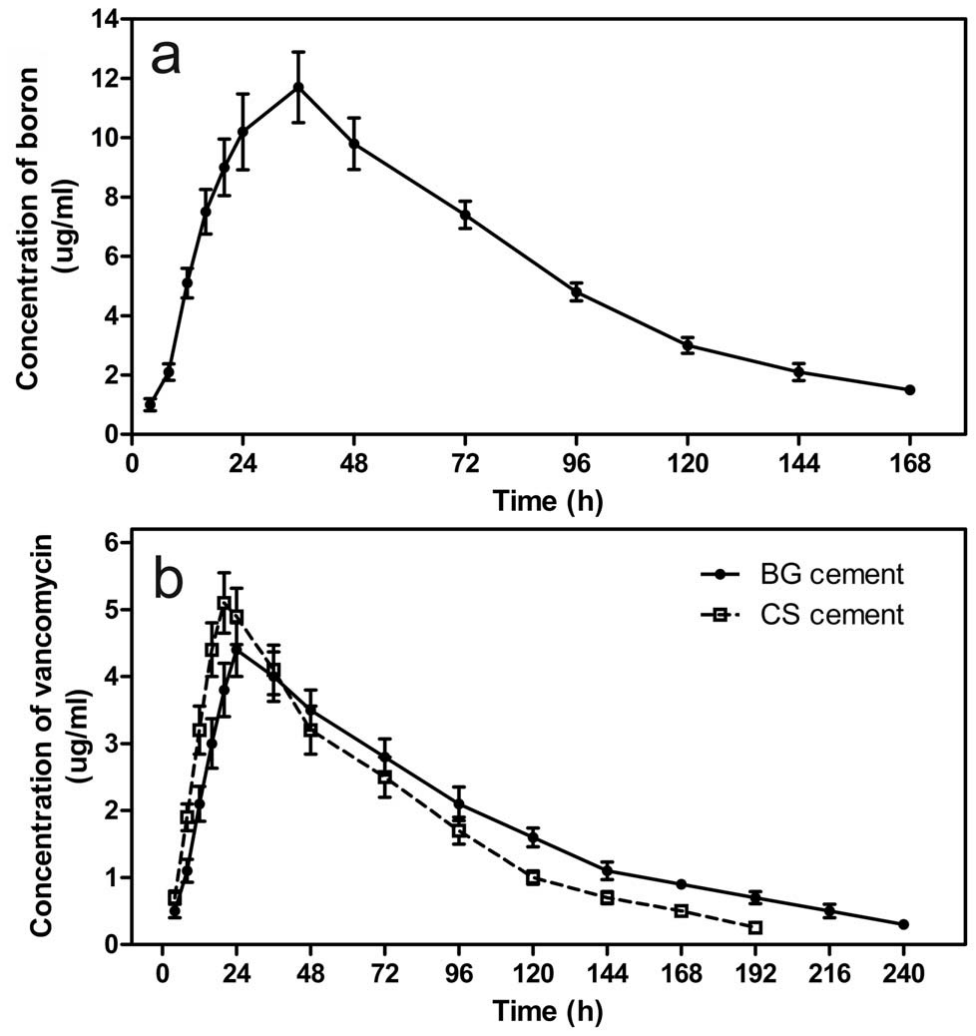
Blood analysis of animals implanted with cements. (a) Boron concentration in the serum of rabbits implanted with vancomycin-loaded borate BG cement as a function of time post-surgery; (b) vancomycin concentration in the serum of rabbits implanted with borate BG cement and CS cement as a function of time post-surgery.

Although there were fluctuations, the white blood cell (WBC) counts and the liver and renal functions at sacrifice showed no measurable differences from preoperative values. The boron concentration in the serum of the animals implanted with vancomycin-loaded BG cement reached a peak at 36 h post-surgery, and gradually decreased thereafter (Fig. 6a). The vancomycin concentration in the serum of the animals implanted with BG cement or CS cement remained at a low level (Fig. 6b). In the Group 4 animals, the vancomycin concentration reached a peak (4.4 µg/mL) at 24 h post-surgery and decreased to a value below the detection limit (0.2 µg/mL) at day 12. In comparison, the concentration reached a peak value of 5.1 µg/mL in the Group 3 animals at 20 h post-surgery and decreased to a value below the detection limit at day 8.

### Radiographic evaluation

**Figure 7:**
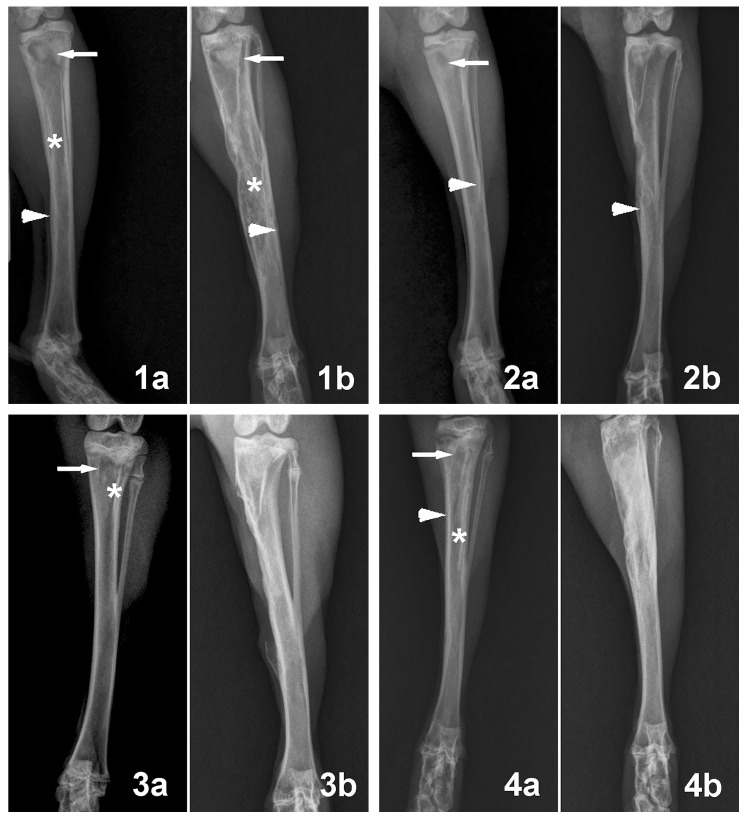
Radiographics analysis of animals before surgery and at 2 months post-surgery. Representative preoperative radiographs showing tibial osteomyelitis in the four different groups (1a, 2a, 3a, 4a), characterized by bone destruction (arrows), periosteal new bone formation (arrowhead) and sequestral bone formation (asterisk). (1b) Postoperative radiographs of Group 1 showed deterioration of osteomyelitis coupled with periosteal new bone formation (arrowhead), destruction of bone (arrows) and sequestral bone formation (asterisk). (2b) Postoperative radiographs of Group 2 showed that the osteomyelitis was partly controlled. (3b, 4b) Postoperative radiographs of Group 3 and Group 4 showed that the osteomyelitis was healed.

**Figure 8:**
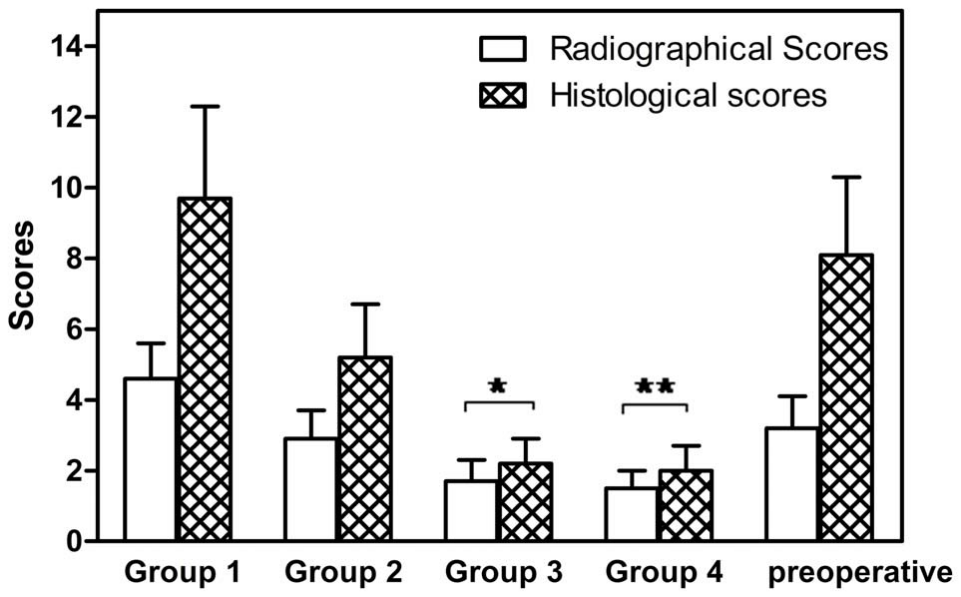
Radiographic scores and histological scores for rabbit tibiae infected with osteomyelitis prior to treatment (pre-operative) and after 8 weeks of treatment in the 4 groups. Group 1: no treatment; Group 2: intravenous injection of vancomycin; Group 3: vancomycin- loaded CS cement; Group 4: vancomycin-loaded borate BG cement. (mean ± SD; *, ** significantly different to the corresponding scores for the other groups, *P*<0.05).

For all the animals used in this study, typical signs of bone infection including SBF, DB, and PNBF were seen in the radiographic images before surgery, and no clear differences were observed among the groups. Two months post-surgery, radiographic images of the tibiae showed significant improvement in the Group 3 and 4 animals (Fig. 7). Only 2 of the 16 animals in each of those two groups showed the presence of osteomyelitis, indicating the capacity of both treatments to cure the bone infection. In comparison, 9 of the 16 animals in Group 2 and all 10 animals in Group 1 still showed signs of osteomyelitis. The numbers of infected animals in Groups 3 and 4 were significantly lower than those in Groups 1 and 2 (Table 1). The mean radiographic scores of the Group 3 and Group 4 animals were also significantly lower than those of Groups 1 and 2 (Fig. 8).

**Table 1:** Comparison of the capacity of the 4 treatment mentods to eradicate MRSA-induced osteomyelitis in rabbit tibiae at 8 weeks post-surgery.

Group	Treatment	Total number	Number eradicated (radiology)	Number eradicated (microbiology)
1	No treatment	10	0	0
2	Intravenous vancomycin	16	7	7
3	Vancomycin-loaded CS cement	16	14	13
4	Vancomycin-loaded BG cement	16	14	14

### Microbiological evaluation

The eradication of osteomyelitis was also evaluated by microbiological testing (Table 1). Two months post-surgery, all 10 animals in Group 1 and 9, 3, and 2 animals, respectively, of the 16 animals in each of Groups 2, 3, and 4 tested positive for MRSA-induced infection by cultivation in BHI bouillon at 37°C for 7 days. The number of animals that tested positive in Groups 3 and 4 was significantly lower than the pre-operative number and the numbers in Groups 1 and 2. However, the difference between the number in Group 3 and in Group 4 was not significant.

### Histological Examination and SEM analysis

**Figure 9:**
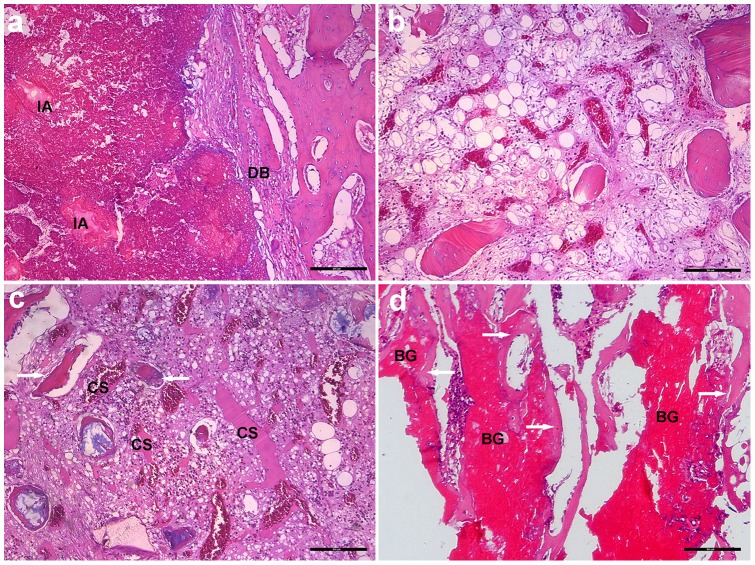
Representative images of H&E stained sections of rabbit tibiae at 2 months post-surgery. (a) Group 1 (no treatment): Typical signs of osteomyelitis, including destruction of bone (DB), intramedullary abscess (IA), with virtually no new bone formation; (b) Group 2 (intravenous injection of vancomycin): no typical signs of osteomyelitis and little new bone formation; (c) Group 3 (vancomycin-loaded CS cement): the CS cement was mostly degraded with only limited new bone formation (white arrow) surrounding it, and some macrophages near the degraded material (CS); (d) Group 4 (vancomycin-loaded borate BG cement): a considerable amount of new bone (white arrow) was formed which was in direct contact with the degraded implant (BG). (Bar  = 200 µm).

Histological evaluation using light microscopy showed clear differences in the appearance of the bone defect area among the four groups of animals (Fig. 9). In Group 1, the defect area was replaced by connective tissue and there were many inflammatory cells in the area; sequestered bone formation and destruction of bone could also be observed (Fig. 9a). In Group 2, no typical signs of osteomyelitis were observed in some defects; some inflammatory cells infiltrated the defect area and there was little new bone formation (Fig. 9b). The implants in Group 3 were almost completely degraded and there was modest new bone formation (Fig. 9c); the presence of some macrophages near the degraded materials indicated the phagocytic process of the host to dissolve the CS cement. Some connective tissue was also found around the newly formed bone. In Group 4, a considerable amount of new bone was formed along with the degradation and conversion of the BG glass in the cement, and the new bone was intimately attached to the surface of the implant or had infiltrated the implant (Fig. 9d).

**Figure 10:**
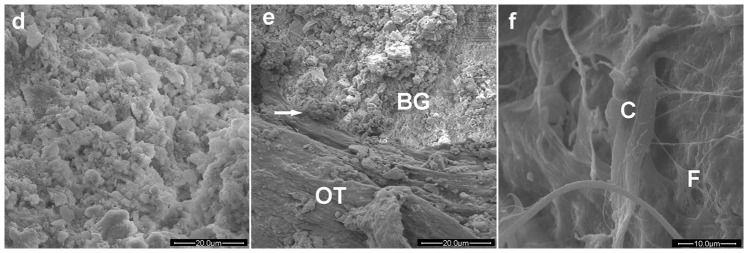
SEM images of the cement after implantation for 8 weeks in rabbit tibiae infected with MRSA-induced osteomyelitis. The BG implant became porous (a), was bonded to osteoid tissues (OT) at the interface (arrow) (b), and some cells (C) and fibrous tissue (F) adhered to the implant (c).

The histological score for Group 4 (2.0±0.8) at 2 months post-surgery was significantly lower than the pre-operative score, and it was the lowest value among the four groups (Fig. 8). SEM images of the defects implanted with the vancomycin-loaded BG cement showed many micro-cavities and pores in the implants, with some cells and fibers growing into them. (Fig. 10a–c).

## Discussion

In general, carriers for local antibiotic delivery in the treatment of osteomyelitis should provide the requisite antibiotic delivery rate, be osteoconductive to enhance bone regeneration after the infection is cured, and have the requisite mechanical strength to support physiological loads [3], [29]. In this study, we evaluated a borate BG cement, composed of borate glass particles, chitosan, citric acid and glucose, as an injectable local antibiotic delivery system. When compared to a commercial calcium sulfate (CS) cement used clinically, the borate BG cement showed a more sustained release of vancomycin *in vitro* and a similar capacity to eradicate MRSA-induced osteomyelitis in a rabbit tibial model *in vivo*. Whereas the CS cement degraded and induced a local inflammatory reaction in the tibial defects, the borate glass particles in the BG cement converted to HA and supported considerable bone regeneration in the defects within the two-month implantation period. Based on these characteristics, the vancomycin- loaded borate BG cement used in the present study has potential for curing osteomyelitis and for regenerating bone after the infection is cured.

The borate BG cement self-hardened when the separate components were mixed together. The setting reactions of the injectable borate BG cement could be divided into two stages. The initial reaction was a chelation reaction between citric acid and calcium in the borate BG, which happened when the powder component and the liquid component were mixed *in vitro*. The second reaction involved transformation of the cement components to HA, which occurred when the cements were placed in an aqueous phosphate solution, such as PBS or body fluid. The setting time of the initial reaction could be modulated by changing the concentration of citric acid and glucose [30], [31]. The initial and final setting times of borate BG cement were 8 and 28 min, respectively, which were longer than those of calcium phosphate cements [32]. This extended setting time could offer the surgeons more time for operation, and it allowed the cements to fill the irregular bone cavity.

In addition, the borate BG cement had an injectability of >90% within the first 3 min after mixing, which was also favorable for clinical use. The strength of the BG cement is appropriate for supporting physiological loads, with a compressive strength, after hardening, of 18.2±2.3 MPa, which is lower than that of other bioactive glass-ceramics [30]. A reduction in strength occurred after immersion of the cement into PBS. However, after immersion in PBS for 7 days, the strength of the cement was still comparable to the highest strength reported for trabecular bone (2−12 MPa). Therefore, the compressive strength of borate BG cement is favorable for application in the repair of osteomyelitis-induced contained bone defects, where adjacent cortical bone could provide mechanical rigidity and support to withstand shear and tensile stress [33].

When placed in an aqueous phosphate solution, borate BGs degrade and convert to an amorphous calcium phosphate (ACP) that subsequently crystallizes to HA. As the glass degrades, calcium ions released from the glass react with phosphate ions from the medium to precipitate ACP (or HA) on the surface of the glass. *In vivo*, the presence of carbonate ions in the body fluid leads to the formation of a carbonate-substituted HA in which a fraction of the phosphate ions are substituted by carbonate ions. The formation of this carbonate-substituted HA leads to a bioactive and osteoconductive material that bonds strongly to bone. Incorporation of collagen and other proteins into the carbonate-substituted HA during the conversion process results in a material with bone-like features. In the present study, immersion of the vancomycin-loaded borate BG cement in PBS *in vitro* apparently resulted in the precipitation of HA on the surface of the cement, because the chitosan bonding phase does not degrade in the absence of biological proteins. However, within 2 months of implantation in rabbit tibial defects, the cement formed an implant that had a good biocompatibility with the host bone and showed the presence of adherent cells and fibers.

The release of vancomycin from both borate BG cement and CS cement into PBS *in vitro* showed the same general profile: a more rapid release initially (first 2 days) followed by a more sustained release thereafter. However, the borate BG cement showed a slower and more sustained release. The release of vancomycin from the borate BG cement also lasted for a longer period when compared to vancomycin-loaded borate glass particles and antibiotic-loaded glass/PMMA composite [12], [34]. In the present study, the free amine groups in the polymeric chains of chitosan were able to interact with negatively-charged groups in the polymers and with polyanions [35]. Consequently, cross-linking could occur between the amine groups of chitosan and phosphate (PO_4_)^3−^ ions in the PBS which could reduce the release rate of vancomycin. The antibiotic activity of vancomycin released from borate BG cement *in vitro* was similar to that of the as-received vancomycin, which indicates the feasibility of the cement preparation process for clinical application. The vancomycin concentrations in the eluents exceeded the MIC for MRSA but was lower than the toxic concentration, so could ensure local effective bactericidal concentration with no inhibition of bone regeneration [36], [37].

The boron and vancomycin concentrations in the serum of the infected rabbits implanted with vancomycin-loaded borate BG cement were well below the toxic levels throughout the implantation period. Concerns about the toxicity of boron and vancomycin released from the borate BG cement can therefore be allayed. Radiography and microbiological tests showed that when loaded with vancomycin, both the borate BG cement and the CS cement were significantly more effective than intravenous injection of vancomycin in eradicating MRSA-induced osteomyelitis in the rabbit tibiae. While the borate BG cement showed a more sustained release of vancomycin *in vitro*, there was no significant difference in the capacity of the two implant materials to eradicate osteomyelitis.

The ability to promote bone formation after curing infection is desirable because osteomyelitis is often associated with bone loss [38]. A considerable amount of new bone was formed in the defect sites implanted with borate BG cement at the end of the two-month implantation. No inflammatory cells were observed in the vicinity of the vancomycin-loaded BG cement, indicating the biocompatibility of that cement *in vivo*. In comparison, the CS cement degraded rapidly and caused local inflammation, a finding in agreement with the reported drawbacks of calcium sulfate *in vivo* [39], [40].

## Conclusions

A vancomycin-loaded biomaterial composed of borate BG particles bonded with chitosan had initial and final setting times of ∼8 and ∼28 min, respectively, rendering the material suitable for use as an injectable cement. With a compressive strength of 18.2±2.3 MPa after hardening, the cement could be used in some load-bearing bone sites. *In vitro*, the borate BG cement showed a sustained release of vancomycin into PBS over a longer period (∼36 days) when compared to a commercial CS cement used clinically (∼28 days). When loaded with vancomycin (8 wt%), the BG cement showed a similar capacity to eradicate MRSA-induced osteomyelitis compared to CS cement and a better capacity compared to intravenous injection of vancomycin. The BG cement also exhibited a good capacity to form new bone in the defect sites. The BG cement is therefore a promising injectable carrier for vancomycin in eradicating osteomyelitis and as an osteoconductive matrix to regenerate new bone after the infection is cured.
